# Mixed application of microbial fertilizers reshapes the tobacco rhizosphere microbiome and enhances metabolic coordination to improve crop quality

**DOI:** 10.3389/fmicb.2025.1726681

**Published:** 2026-01-15

**Authors:** Xianjun Lai, Songlin Hou, Siqi Liu, Wenyou Zhang, Zhengsong Peng, Jie Yang, Lang Yan, Xianjun Wang

**Affiliations:** 1 Panxi Crops Improvement Key Laboratory of Sichuan Province, Xichang University, Liangshan, China; 2 College of Agriculture Sciences, Sichuan Agricultural University, Chengdu, China; 3 China Tobacco Sichuan Industrial Co., Ltd., Chengdu, China

**Keywords:** flue-cured tobacco, microbial fertilization, rhizosphere microbiome, secondary metabolism, chemical coordination

## Abstract

Sustainable management of flue-cured tobacco requires a careful balance among productivity, chemical composition, and soil ecological function, which are often disrupted by excessive chemical fertilization. This study aimed to elucidate how microbial fertilization regulates plant performance, chemical coordination, and rhizosphere microbial structure under field conditions. A two-year factorial field experiment was conducted in Sichuan, China, using a *Bacillus*-based plant growth-promoting microorganism (PGPM) and a commercial microbial consortium (Xi⋅Weifeng), applied individually or in combination at gradient doses. Agronomic traits, cured-leaf chemical composition, secondary metabolites, and rhizosphere bacterial communities were comprehensively analyzed using multivariate statistics, network correlation analysis, and structural equation modeling (SEM). Moderate PGPM application (27 kg⋅ha^–1^) significantly increased plant height (8.6%), internode length (15.3%), and leaf width (7.8%) at the vigorous growth stage. Co-application further enhanced leaf expansion (9.7%) and improved chemical coordination, maintaining optimal sugar/nicotine (8–12) and N/nicotine (0.7–1.0) ratios. Chlorogenic acid (18.8 mg⋅g^–1^) and neochlorogenic acid (2.7 mg⋅g^–1^) were markedly elevated under the A27B54 treatment. Rhizosphere bacterial diversity peaked under co-application, with *Bacillus*, *Rhizobiales*, and *Sphingomonas* emerging as key taxa positively associated with both metabolic and agronomic improvements. SEM demonstrated that fertilization effects on leaf quality were mediated indirectly through microbial community restructuring and metabolite modulation. Microbial fertilizer co-application enhances tobacco performance by promoting rhizosphere microbial diversity and functional coordination, which in turn improves metabolic balance and nutrient-use efficiency. These findings highlight a soil microbiome-mediated pathway linking fertilization strategy to crop physiological and chemical responses, providing mechanistic insights for sustainable fertilization management.

## Introduction

Flue-cured tobacco quality is a central determinant of the competitiveness and economic value of the tobacco industry, and its overall performance depends not only on yield but also on the coordination of chemical constituents and sensory traits (Hui and Hao, 2011; Li J. et al., 2024). In conventional production systems, long-term and excessive application of chemical fertilizers has promoted biomass accumulation but often caused imbalances in key chemical indices such as the sugar-to-alkaloid ratio and the potassium-to-chlorine ratio, thereby weakening combustibility, aroma, and overall harmony (Huan et al., 2024; Pace et al., 2020). In addition, overreliance on chemical fertilizers has led to declines in soil organic matter, reduced nutrient cycling efficiency, and deterioration of rhizosphere microbial diversity, all of which constrain nutrient uptake and ultimately limit improvements in leaf quality (Shen et al., 2022). Against the backdrop of high-end raw tobacco production, balancing yield, quality, and soil health has become a pressing challenge for the tobacco production system.

In recent years, microbial fertilizers have attracted considerable attention as environmentally friendly inputs, with increasing evidence demonstrating their potential to enhance crop quality. Functional microbes can influence quality formation through multiple pathways, including nutrient supply, metabolic regulation, and rhizosphere restructuring (Feng et al., 2023; Wang X. et al., 2025). At the nutrient level, nitrogen-fixing, phosphate-solubilizing, and potassium-mobilizing bacteria improve nitrogen assimilation efficiency and carbon–nitrogen balance, thereby optimizing the sugar–alkaloid balance of flue-cured tobacco leaves (Gao et al., 2016; Shi et al., 2024). At the metabolic level, microbial fertilization has been shown to activate the phenylpropanoid pathway and tricarboxylic acid (TCA) cycle, leading to enhanced accumulation of chlorogenic acid, solanesol, and organic acids such as malic and citric acid, which are closely linked to aroma, leaf color, and burning properties (Li W. et al., 2024; Zhong et al., 2025). At the rhizosphere level, microbial fertilizers reshape community structures by enriching functional groups such as Proteobacteria and Actinobacteria, and these shifts are strongly correlated with key chemical indices of tobacco quality (Wang S. et al., 2025). Moreover, certain strains can induce systemic resistance (ISR) via jasmonic acid (JA) and ethylene (ET) signaling, which not only enhance plant defense but also cross-regulate the biosynthesis of quality-related secondary metabolites (Jain et al., 2016). Collectively, these findings outline a multilayered “rhizosphere-metabolite-quality” axis that provides mechanistic evidence for the quality-enhancing potential of microbial fertilization.

Despite these advances, significant gaps remain in both research and application. Most studies have focused on single strains or products, with little attention to the synergistic effects of different microbial fertilizers across dosage gradients. Existing work often emphasizes yield or isolated quality indices, whereas the integrated relationships among chemical coordination, sensory evaluation, and secondary metabolite profiles remain poorly resolved. Furthermore, field-scale applications are complicated by the variability of soil microbial communities across ecological contexts, which contributes to inconsistent outcomes in practice (Zhang et al., 2022). These limitations hinder the identification of optimal fertilizer combinations under specific production conditions and restrict the translation of microbial fertilization from experimental trials to large-scale deployment. In the context of premium tobacco production, the lack of field-based, systematic evidence has become a major bottleneck preventing the wider adoption of microbial fertilizers.

To address these challenges, this study was conducted in the premium flue-cured tobacco production base of Puge County, Liangshan, Sichuan Province, China. We systematically evaluated the individual and combined effects of two novel microbial fertilizers applied at different concentrations, focusing on their impacts on agronomic traits, chemical coordination, sensory quality, secondary metabolites, and rhizosphere microbial community composition. By integrating dose-response and synergy analyses, the study elucidates key mechanisms by which microbial fertilizers improve flue-cured tobacco quality and identifies the optimal fertilization strategy for local production conditions. These findings not only provide a scientific basis for premium tobacco cultivation but also offer a broader paradigm for expanding microbial fertilization into other specialty crops, highlighting both theoretical and practical significance.

## Material and methods

### Growth chamber pot experiment and sterile inoculation assays

A growth chamber pot experiment was conducted to evaluate the plant growth-promoting potential of two core PGPM strains, *Bacillus subtilis* and *B. licheniformis*. Both strains were revived from glycerol stocks and cultured in LB broth at 30 °C and 200 rpm for 16–18 h. Cells were harvested by centrifugation, washed, and resuspended in sterile PBS to a final concentration of 1 × 10^8^ CFU⋅mL^–1^ for inoculation. Tobacco seedlings at the 4–5 true-leaf stage were transplanted into 12 cm pots filled with a peat:perlite:vermiculite substrate (3:1:1, v/v/v), with one plant per pot. Immediately after transplanting, 20 mL of bacterial suspension was applied to the rhizosphere, followed by a second inoculation on day 7; control plants received an equal volume of sterile PBS. Plants were maintained for 60 days under controlled growth chamber conditions (16 h light/8 h dark; 250 μmol m^–2^ s^–1^ light intensity; 26 °C/22 °C day/night; 60% relative humidity). At the early flowering stage, agronomic traits including plant height, leaf number, maximum leaf length, and maximum leaf width were recorded. Root traits were documented at harvest by gently removing the substrate, rinsing roots with deionized water, and imaging the entire root system.

To assess the contribution of microbial metabolites independent of bacterial colonization, representative growth-related metabolites in both the self-prepared PGPM fertilizer and the commercial product Xi⋅Weifeng were quantified, including indole-3-acetic acid (IAA), ACC deaminase, siderophores, surfactin, and iturin. IAA content was measured using the Salkowski colorimetric assay (Gordon and Weber, 1951). ACC deaminase activity was determined following Glick (1995) by quantifying the production of α-ketobutyrate (Penrose and Glick, 2003). Siderophore levels were measured using the Chrome Azurol S (CAS) assay (Schwyn and Neilands, 1987). Surfactin and iturin were extracted with methanol and quantified via HPLC according to the protocol of Meena and Kanwar (2015). Metabolite data were reported as concentrations or relative activities.

To further determine whether these metabolites were capable of triggering growth promotion, sterile plant inoculation assays were performed. Tobacco seeds were surface-sterilized with 70% ethanol for 30 s and 2.5% sodium hypochlorite for 8 min, washed five times with sterile water, and germinated on sterile MS agar medium under controlled conditions (25 °C; 16 h light/8 h dark). Seedlings at the three-leaf stage were transferred to sterile glass jars containing autoclaved vermiculite. Cell-free filtrates of PGPM fertilizer and Xi⋅Weifeng were prepared by passing the suspensions through a 0.22-μm membrane filter, and 5 mL of each filtrate was applied per plant; sterile water served as the control. After 20 days of growth, plant height, leaf number, maximum leaf length, and leaf width were measured to evaluate metabolite-mediated effects in the absence of microbial colonization.

### Field experimental design

The field experiment was conducted over two consecutive growing seasons at the Guobao (Kuan-Zhai) premium flue-cured tobacco production base in Puge County, Sichuan Province, China (27°13′-27°30′ N, 102°26′-102°46′ E; ∼2,000 m a.s.l.). The region is characterized by a subtropical plateau climate, with a mean annual temperature of 16.8 °C, annual precipitation of 1,169.8 mm, and annual sunshine duration of 2,094.7 h. The soil type is sandy loam, with initial physicochemical properties as follows: pH 5.5–7.0, organic matter 39.93 g⋅kg^–1^, total N 0.77 g⋅kg^–1^, available N 130.18 mg⋅kg^–1^, total P 0.95 g⋅kg^–1^, available P 12.82 mg⋅kg^–1^, total K 17.01 g⋅kg^–1^, and available K 96.44 mg⋅kg^–1^. The flue-cured tobacco (*Nicotiana tabacum* L.) cultivar Yunyan 85 was used, and the experimental plots (∼10 mu, ca. 0.67 ha) were contiguous fields managed by the same farmer, with soil chloride concentrations <30 mg⋅kg^–1^.

Two microbial fertilizers were tested. The first was a plant growth-promoting microbial (PGPM) inoculant prepared in-house, consisting of a mixed consortium of *Bacillus subtilis*, *Bacillus licheniformis*, and *Paenibacillus polymyxa* at a biomass ratio of 2:2:1, with a viable cell count of 5.0 × 10^8^ CFU⋅g^–1^. The second was a commercial microbial fertilizer, Xi⋅Weifeng, formulated from functional strains belonging to *Bacillus* spp., *Pseudomonas* spp., and *Streptomyces* spp., combined with organic matter, amino acids, and trace element carriers.

The experimental design followed a two-factor, three-level factorial arrangement. Each fertilizer was applied at three rates (0, 27, and 54 kg⋅ha^–1^), resulting in nine treatment combinations. Treatments were arranged in a randomized block design with three replicates, and each plot contained 40 plants. A uniform application of organic fertilizer (900 kg⋅ha^−1^) was used as the basal fertilizer across all plots. Microbial fertilizers were first applied 1 week after transplanting, diluted with 3000 L⋅ha^–1^ water, and administered via a combination of foliar spraying and root drenching. Subsequent applications were repeated at 3-week intervals for a total of four times, always scheduled either before 10:00 a.m. or after 4:00 p.m. on cloudy days.

### Measurements of agronomic traits, chemical constituents, secondary metabolites, and comprehensive leaf quality

Agronomic traits were assessed at both the vigorous growth stage and the maturity stage. Within each plot, 15 uniformly growing plants were randomly selected to record plant height, stem girth, maximum leaf length, maximum leaf width, and total leaf number. Measurements followed the national standard Agronomic Trait Survey Methods for Tobacco (YC/T 142–2010).

After harvest and curing, leaf samples for chemical composition analysis were collected according to the current flue-cured tobacco grading standards based on leaf position and color. From each treatment, one representative grade was selected from the upper (B2F), middle (C3F), and lower (X2F) stalk positions and 1 kg of leaves was sampled for each grade. Samples were dried at 60°C for 3–4 h, destalked, ground, passed through a 60-mesh sieve, and then sealed for subsequent analyses.

The chemical composition of flue-cured tobacco leaves was determined following standard procedures. Total alkaloids (expressed as nicotine) were measured by ultraviolet spectrophotometry after alkaline distillation, following CORESTA RM-12 (Determination of Alkaloids in Cigarette Smoke Condensates, September 1968) with minor adaptations for leaf samples. Total chlorine (Cl) was analyzed by hot-water extraction followed by volumetric titration using the AOAC Official Method 915.01 (AOAC, 2000). Total nitrogen (N) content was quantified using the Kjeldahl method according to ISO 1871:2009 (ISO, 2009). Total potassium (K) was determined by flame photometry following the Chinese Tobacco Industry Standard YC/T 173-2003 (State Tobacco Monopoly Administration, 2003). Total sugars were measured by the ethanol extraction-anthrone colorimetric method following recent practice in tobacco leaf chemistry (e.g., Yin et al., 2024), and reducing sugars were determined by the 3,5-dinitrosalicylic acid (DNS) colorimetric method, as applied to tobacco extracts in recent studies (e.g., Mai et al., 2024). Chemical composition evaluation was performed according to the Technical Standard for Sensory Quality Evaluation of Single Tobacco Leaves (QJ/02.J.001-2016.A), focusing on eight key indices: total alkaloids, total sugars, potassium, chlorine, the sugar-to-alkaloid ratio (sugar/nicotine), the nitrogen-to-alkaloid ratio (N/nicotine), the potassium-to-chlorine ratio (K/Cl), and the difference between total and reducing sugars (Δsugers).

To further characterize the secondary metabolite profile of cured leaves, Fourier-transform near-infrared spectroscopy (FT-NIR) was conducted using an MPA spectrometer. Spectral analysis enabled quantification of carotenoids, maillard reaction products, phenylalanine-derived degradation products, cembranoid derivatives, and neophytadiene.

Comprehensive quality evaluation of cured leaves was conducted following the principles outlined in GB/T 18771.4-2015 (Tobacco Terminology, Part 4: Quality and Testing). Three dimensions were considered: chemical coordination (20%), appearance quality (15%), and sensory quality (60%). A weighted contribution was assigned to different leaf positions: upper leaves (B) 40%, middle leaves (C) 45%, and lower leaves (X) 15%. Final evaluations integrated chemical composition data, appearance indices, and professional sensory panel scores to generate an overall quality assessment for each treatment.

### Soil sampling and DNA extraction

Rhizosphere soil samples were collected at the maturity and curing stages using an “S-shaped” five-point sampling method. For each treatment, multiple subsamples were randomly taken, thoroughly homogenized into a composite sample, and immediately sealed under low temperature. Samples were transported to the laboratory and temporarily stored at 0 °C prior to DNA extraction. Total genomic DNA from the rhizosphere soil was extracted using the E.Z.N.A.^®^ Soil DNA Kit (Omega Bio-Tek, Norcross, GA, USA) following the manufacturer’s instructions. The extracted DNA was subsequently used for amplification and high-throughput sequencing.

The bacterial 16S rRNA gene was amplified targeting the V3-V4 hypervariable regions with the primer set 338F (5′-ACTCCTACGGGAGGCAGCAG-3′) and 806R (5′-GGACTACHVGGGTWTCTAAT-3′). PCR reactions contained 10 ng of template DNA and were carried out under the following conditions: an initial denaturation at 95 °C for 3 min; 27 cycles of denaturation at 95 °C for 30 s, annealing at 55 °C for 30 s, and extension at 72 °C for 45 s; followed by a final extension at 72 °C for 10 min. PCR products were purified, quantified, pooled at equimolar concentrations, and subjected to paired-end sequencing on the Illumina NovaSeq 6000 platform (Illumina Inc., San Diego, CA, USA) at Biomarker Technologies Corporation (Beijing, China).

### Microbial diversity data processing

Raw reads were processed in QIIME2 for demultiplexing, quality control, and denoising, and amplicon sequence variants (ASVs) were inferred using the DADA2 algorithm (Bolyen et al., 2019; Callahan et al., 2016). Very low-abundance features (total counts < 2 across all samples) were filtered out prior to downstream analyses. Taxonomic assignment of ASVs was performed with the QIIME2 naïve Bayes classifier trained on the SILVA reference database (release 138.1) with a confidence threshold of 70% (Quast et al., 2012).

Alpha-diversity indices were computed in QIIME2 and visualized in R (v4.2.2) using ggplot2. Rarefaction curves were generated to assess sequencing depth and sampling completeness. Group differences in alpha diversity were evaluated using the Kruskal-Wallis test; when significant, pairwise Wilcoxon rank-sum tests were conducted with false discovery rate (FDR) correction.

Beta diversity was calculated using Bray-Curtis dissimilarity and weighted UniFrac distances. Ordinations were visualized by principal coordinates analysis (PCoA) implemented in the vegan package in R. Differences in community composition among groups were tested by permutational multivariate analysis of variance (PERMANOVA; adonis, 999 permutations) and further assessed with analysis of similarities (ANOSIM), with statistical significance set at *p* < 0.05 (Oksanen et al., 2014).

Linear discriminant analysis effect size (LEfSe) was used to identify differential microbial taxa among fertilizer treatments. The genus-level abundance table was used as input. The Kruskal-Wallis rank-sum test (α = 0.05) was applied to detect significantly different taxa across groups, followed by pairwise Wilcoxon rank-sum tests between subclasses. LDA was then performed to estimate the effect size of each discriminative feature, and taxa with an LDA score >4.0 were retained. Multiple testing correction was performed using the false discovery rate (FDR). LEfSe analysis was conducted in Galaxy platform^1^ and cross-validated with the lefser (v1.0) package in R.

The network was constructed using Python (v3.11.7). Biomarkers identified by LEfSe were selected, and their relative abundances were correlated with agronomic and chemical traits using Spearman’s rank correlation test. Only significant correlations (*p* < 0.05 after FDR adjustment) were retained for visualization. Visualization was generated using Matplotlib (v3.6) and refined with Gephi (v0.10.1) for layout optimization.

Structural equation modeling (SEM) was applied to quantify the direct and indirect effects of fertilizer treatments, microbial communities, and metabolites on tobacco agronomic and quality traits. Fertilizer dose and type were used as exogenous variables, while microbial community composition (PC1 scores from PCoA), metabolite profiles (PC1 scores from PCA), and agronomic/chemical traits were treated as endogenous variables. The piecewise SEM framework was implemented using the piecewiseSEM package in R. Path coefficients were estimated with linear mixed models, and model fit was evaluated using Fisher’s C statistic and AIC criteria. Significant and marginally significant paths were retained to construct the final SEM, which was visualized using the semPlot package.

### Statistical analysis

All analyses were performed in R. For agronomic traits, chemical indices, and metabolite contents, a two-way ANOVA was fitted with fertilizer type (PGPM vs. Xi⋅Weifeng) and application rate (0, 27, 54 kg⋅ha^–1^) as fixed factors and their interaction. When the overall *F*-test was significant (α < 0.05), pairwise mean separation was conducted using Tukey’s honestly significant difference (HSD). Pearson correlation analysis was used to explore associations among chemical composition metrics, sensory quality scores, and microbial diversity indices; where multiple correlations were evaluated, false discovery rate (FDR)-adjusted *p*-values were reported. Unless stated otherwise, data are presented as mean ± standard error (SE) based on plot-level replicates. Figures were prepared in R with ggplot2 package.

## Result

### Pot experiment validation of PGPM strains prior to field application

Before the field experiment, a laboratory pot trial was conducted to validate the plant growth-promoting potential of the two core PGPM strains, *B. subtilis* and *B. licheniformis*. Inoculation with *B. subtilis* increased plant height from 76.2 ± 6.1 cm in the control to 82.4 ± 5.3 cm, accompanied by a slight increase in leaf number (17.6 ± 1.2 vs. 17.1 ± 1.3) while maximum leaf length remained comparable between treatments (61.8 ± 3.4 vs. 61.2 ± 4.1 cm) and maximum leaf width showed a slight reduction (24.6 ± 2.0 vs. 25.8 ± 3.1 cm). *B. licheniformis* exhibited a milder but consistent effect, increasing plant height from 75.1 ± 5.8 cm to 78.3 ± 5.0 cm, with similar leaf size parameters between treatments. Both strains markedly stimulated root development, producing longer primary roots and greater lateral root density (Supplementary Figure 1). Overall, seedlings inoculated with either strain exhibited a taller plant stature, a more robust stem base, and a more developed root system, indicating the potential of *Bacillus*-based PGPMs in promoting tobacco growth.

To determine whether these growth responses were driven by the direct action of microbial metabolites or were primarily induced by indirect ecological effects arising from strain colonization and subsequent rhizosphere microbiome restructuring, we quantified representative metabolites including IAA, ACC deaminase, siderophores, surfactin and iturin in the self-prepared PGPM fertilizer and the commercial product Xi⋅Weifeng (Supplementary Table 1). Although both microbial fertilizers contained representative hormone-related, nutrient-mobilizing and antimicrobial metabolites, sterile plant inoculation assays showed that direct application of cell-free filtrates did not significantly enhance any growth indicators, indicating that metabolite-mediated direct promotion was limited. Based on these findings, we inferred that the growth-promoting effects of microbial fertilizers were more likely attributable to strain colonization, proliferation, and interactions with native microbial communities within the rhizosphere. Guided by this, we subsequently carried out field-scale fertilization experiments together with parallel analyses of the rhizosphere microbiome, aiming to systematically evaluate the combined impacts of microbial fertilizers on tobacco growth, quality formation, and rhizosphere ecological processes.

### Stage-specific effects of microbial fertilizers on agronomic traits of flue-cured tobacco

During the vigorous growth stage, significant differences in agronomic traits were observed among treatments (Supplementary Table 2). Plant height in the control (A_0_B_0_) was 119.11 cm in average, whereas application of PGPM at 27 kg⋅ha^–1^ (A_27_B_0_) increased height to 129.33 cm, representing an 8.6% improvement (*p* < 0.05). Combined application with Xi⋅Weifeng also significantly enhanced height (A_27_B_27_, 128.78 cm; A_27_B_54_, 129.22 cm). Internode length was significantly extended by PGPM at 27 kg⋅ha^–1^ (5.11 in A_27_B_0_ vs. 4.43 cm in control, *p* < 0.05). Maximum leaf width reached 32.11 cm under A_27_B_0_, an increase of 7.8% compared with the control (29.78 cm), whereas excessive PGPM (A_54_B_0_) reduced leaf width to 28.22 cm (*p* < 0.05), suggesting a dose-dependent inhibitory effect. Other traits, including leaf number (17.89–18.89), stem girth (12.31–12.72 cm), and maximum leaf length (83.78–86.78 cm), showed no significant differences among treatments.

At maturity, responses were most evident in plant height and leaf width. Plant height in the control was 109.11 cm in average, while PGPM at 27 kg⋅ha^–1^ (A_27_B_0_) and its combination with Xi⋅Weifeng (A_27_B_27_) significantly increased height to 118.44 cm and 116.89 cm, respectively (both *p* < 0.05). Internode length increased from 6.01 cm in the control to 6.42–6.62 cm across all fertilizer treatments (*p* < 0.05), indicating enhanced sensitivity of stem elongation to microbial intervention at this stage. Maximum leaf width was most improved under Xi⋅Weifeng at 27 kg⋅ha^–1^ (36.56 cm vs. 33.33 cm in control, *p* < 0.05), whereas PGPM at 54 kg⋅ha^–1^ reduced width to 31.78 cm (*p* < 0.05), confirming a high-dose inhibitory effect.

**FIGURE 1:**
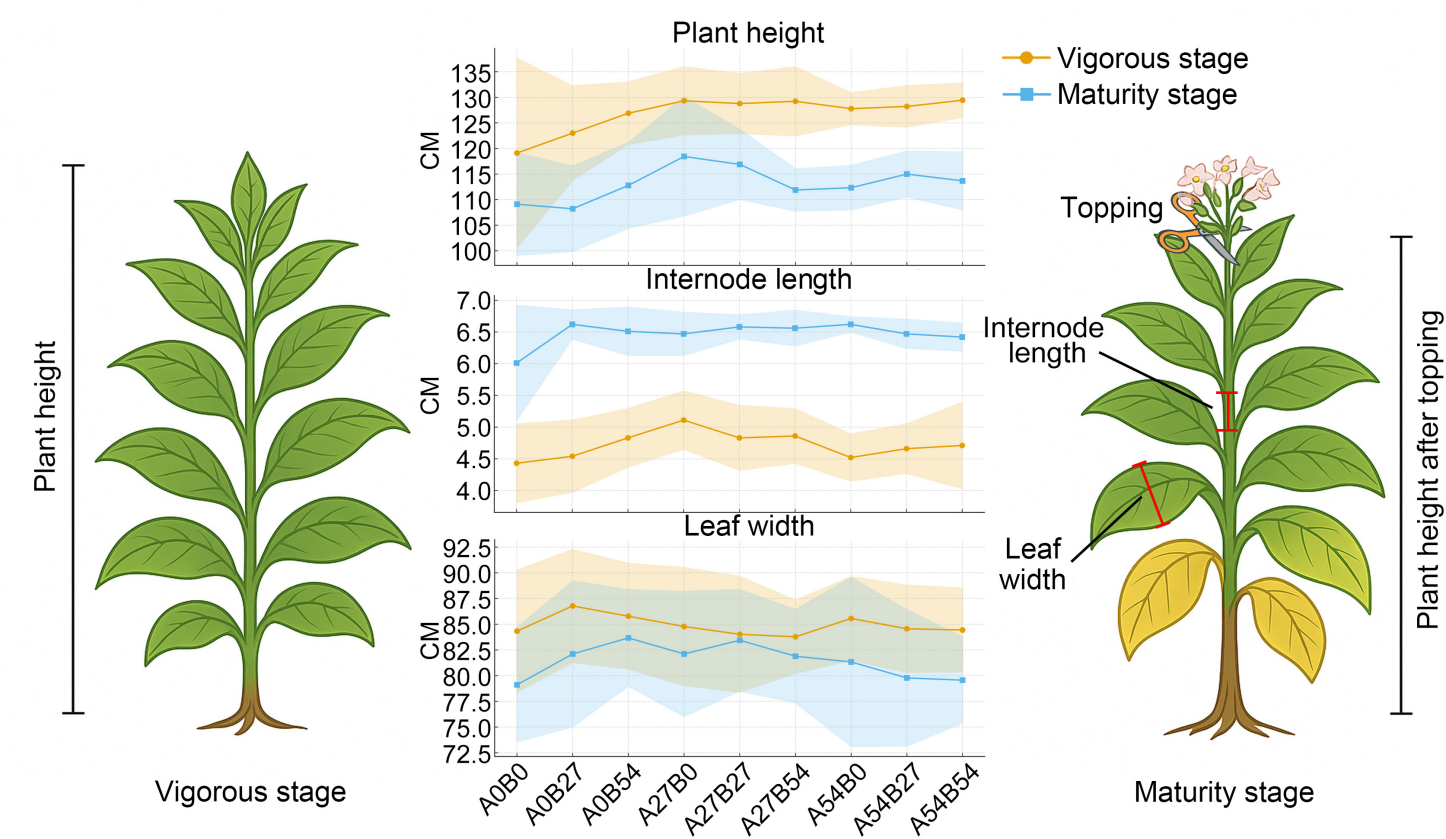
Effects of different fertilization treatments on agronomic traits of flue-cured tobacco. Comparisons of plant height, internode length, and leaf width of flue-cured tobacco under different treatments at the vigorous growth stage (orange) and maturity stage (blue). The schematic diagrams on the left and right illustrate the measurement positions of plant height, internode length, and leaf width.

Taken together, as shown in Figure 1 and Supplementary Figure 2, PGPM at 27 kg⋅ha^–1^ consistently promoted plant elongation throughout the growing season, with significant increases in height (+8.6%), internode length (+15.3%), and leaf width (+7.8%) during the vigorous stage, and maintained height advantage (+8.5%) at maturity. Xi⋅Weifeng, by contrast, primarily enhanced leaf expansion at maturity (+9.7%). Notably, PGPM at 54 kg⋅ha^–1^ caused a reduction in leaf width in both stages, suggesting that excessive application may suppress lateral leaf growth. Overall, PGPM at moderate levels is more suitable for enhancing plant stature and internode elongation, whereas Xi⋅Weifeng at appropriate levels contributes to improved leaf appearance quality.

### Effects of novel fertilizer treatments on chemical constituents of cured tobacco leaves

Fertilizer treatments markedly influenced the chemical composition and quality of cured tobacco leaves (Supplementary Table 3). In upper leaves (B2F), nicotine increased from 3.73% in the control (A_0_B_0_) to 4.09% under Xi⋅Weifeng at 27 kg⋅ha^–1^ (A_0_B_27_). High-dose Xi⋅Weifeng (A_0_B_54_) raised total sugar to 40.6% and the sugar-to-nicotine ratio to 15.55 but compromised coordination with a higher nitrogen-to-nicotine ratio (0.92). The highest quality score was obtained under A_27_B_54_ (69.88, +30% vs. control), followed by A_54_B_27_ (67.08). In middle leaves (C3F), fertilization generally reduced nicotine (4.26% in control vs. 2.20% in A_0_B_54_) and markedly improved sugar-to-nicotine ratios (6.45 in control vs. 15.21 in A_0_B_54_), with comprehensive scores peaking in A_27_B_0_ (72.50, +40.7%), A_54_B_0_ (71.18), and A_0_B_54_ (68.88). In lower leaves (X2F), quality improvement was most pronounced, with A_27_B_54_ and A_54_B_27_ scored exceeding 75.3 (+39%) compared to the control scored 54.03. Nicotine was significantly higher under A_54_B_54_ (1.77%) and A_27_B_54_ (1.75%) than in the control (1.38%), while the sugar-to-nicotine ratio decreased markedly from 22.72 in control to 16.24 (A_54_B_54_) and 13.95 (A_27_B_54_), indicating improved coordination.

**FIGURE 2:**
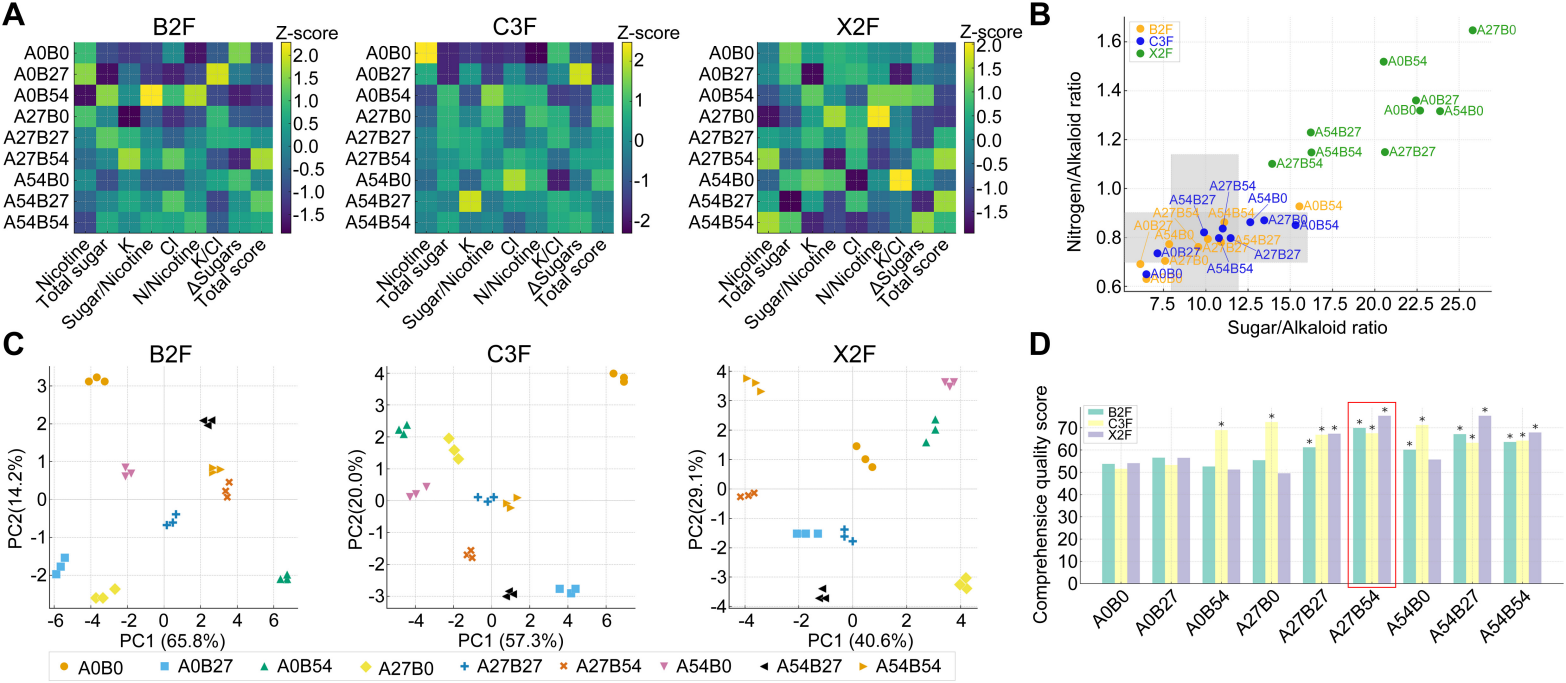
Integrated effects of different fertilizer treatments on the chemical composition of cured tobacco leaves. **(A)** Heatmap showed distribution of major chemical constituents (columns, standardized as Z-scores) across treatments (rows) in upper (B2F), middle (C3F), and lower (X2F) leaves. ΔSugers represented reducing sugar-total sugar difference. **(B)** Principal component analysis (PCA) based on all chemical indicators across leaf positions. The two-dimensional clustering illustrates overall chemical profiles, with clear separations among treatments. **(C)** Sugar-to-nicotine versus nitrogen-to-nicotine ratio plot with the shaded area representing the ideal coordination zone (sugar/nicotine ratio: 8–12; N/nicotine ratio: 0.7–1.0). **(D)** Bar chart comparing composite scores across upper, middle, and lower leaves under different treatments. Significant differences (*p* < 0.05) are indicated by “*”.

Integrating results across all leaf positions, microbial fertilizers markedly improved both chemical coordination and overall quality of cured tobacco leaves (Supplementary Table 2). The heatmap (Figure 2A) clearly revealed distinct regulatory patterns of chemical constituents among treatments, with mixed applications (e.g., A_27_B_54_, A_54_B_27_) showing concurrent enhancements or optimizations across multiple key indices. PCA analysis (Figure 2B) further demonstrated that, compared with either the control or single-factor applications, mixed treatments clustered more closely together and were distinctly separated from the control in the two-dimensional space, indicating that mixed fertilization shaped a more stable and coordinated chemical profile at the integrative level. With respect to coordination indices, the sugar-to-nicotine and nitrogen-to-nicotine ratios are considered critical parameters for assessing the chemical balance of tobacco leaves, and their combined evaluation provides a more comprehensive measure than either parameter alone. As shown in Figure 2C, mixed treatments generally shifted toward the ideal coordination zone (sugar/nicotine ratio: 8–12; N/nicotine ratio: 0.7–1.0) in both upper and middle leaves, with A_27_B_54_ and A_54_B_27_ located closest to the desirable “low nitrogen-to-nicotine and moderate sugar-to-nicotine” range. This suggests that such combinations not only avoided the extremes of excessively high sugar-to-nicotine ratios or overly reduced nicotine induced by single applications, but also maintained superior chemical coordination and sensory potential. Finally, the advantage of mixed fertilization was reinforced by the comprehensive quality scores (Figure 2D), where PGPM at 27 kg⋅ha^–1^ combined with Xi⋅Weifeng at 54 kg⋅ha^–1^ (A_27_B_54_) achieved the highest or near-highest scores in upper (69.88), middle (67.45), and lower (75.35) leaves, emerging as the optimal treatment that consistently enhanced both coordination and overall quality across leaf positions.

### Effects of fertilizer treatments on secondary metabolites in different leaf positions

In the upper leaves (B2F), mixed fertilization produced stronger metabolic enhancements than single treatments. A_27_B_27_ achieved the highest neochlorogenic acid (NCGA, 2.24 mg⋅g^–1^) and significantly increased dichloromethane-soluble compounds (3.95%), while A_54_B_54_ was characterized by marked organic acid accumulation (malic acid 29.3 mg⋅g^–1^, citric acid 1.43 mg⋅g^–1^). In contrast, single treatments such as A_54_B_0_ and A_0_B_54_ improved only specific metabolites (phenolic acids or neophytadiene) but showed limited overall coordination. In the middle leaves (C3F), A_27_B_27_ and A_54_B_54_ were most effective, elevating chlorogenic acid (CGA, 19.83 mg⋅g^–1^) and dichloromethane-soluble compounds (4.40%), respectively. Single treatments provided narrower effects that A_0_B_54_ increased malic acid (24.6 mg⋅g^–1^) but reduced NCGA, whereas A_54_B_0_ mainly enhanced citric acid (1.36 mg⋅g^–1^) without notable gains in phenolics. In the lower leaves (X2F), the effects were most pronounced. A_27_B_54_ produced the highest chlorogenic (18.80 mg⋅g^–1^) and NCGA (2.71 mg⋅g^–1^) while maintaining moderate organic acids, whereas A_54_B_27_ strongly promoted organic acid accumulation (malic acid up to 56.5 mg⋅g^–1^, citric acid 2.15 mg⋅g^–1^). Single treatments mainly boosted citric acid but lacked balanced phenolic improvements.

**FIGURE 3:**
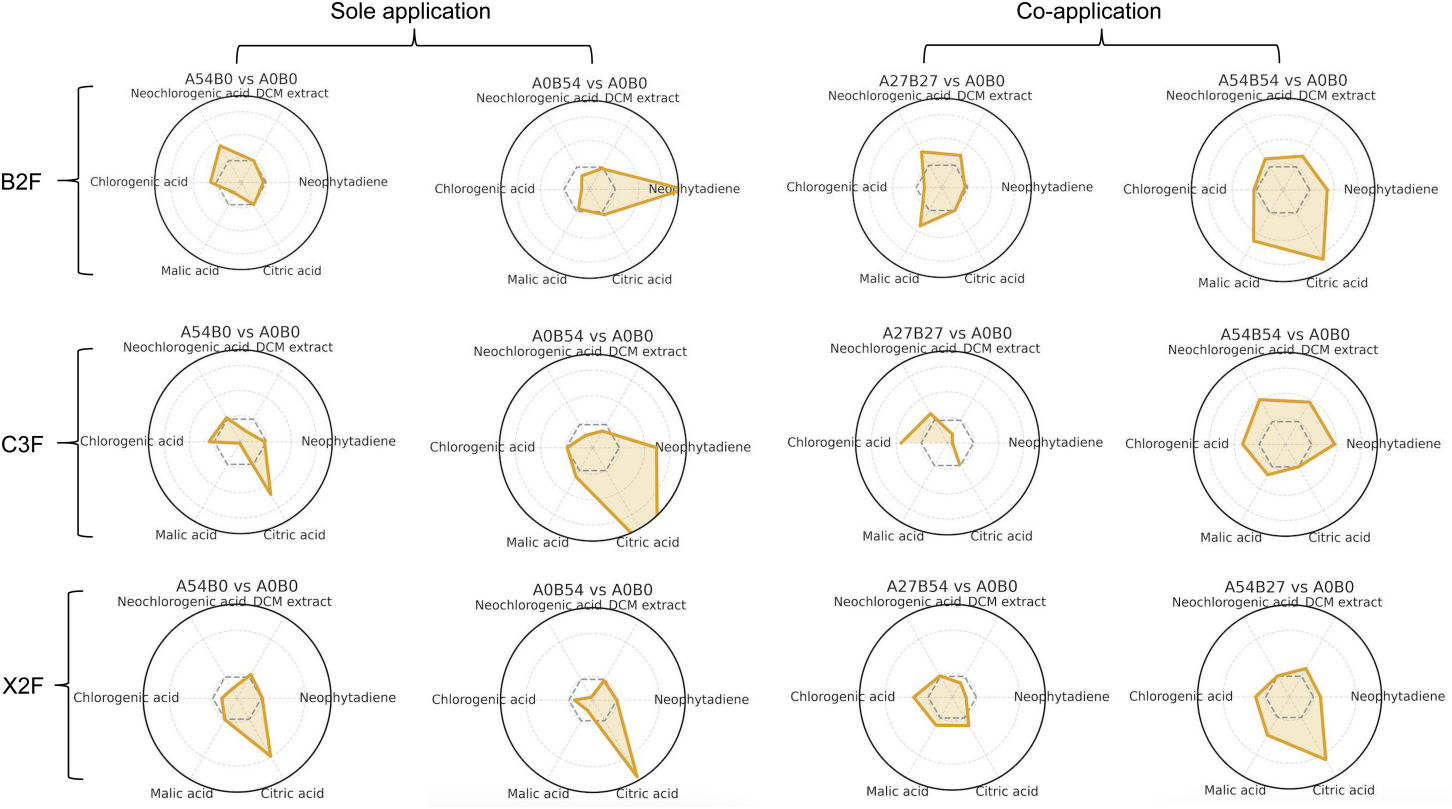
Radar plots of secondary metabolites in upper (B2F), middle (C3F), and lower (X2F) leaves under different fertilizer treatments. Each panel represents one treatment vs. control, with gray dashed lines denoting A_0_B_0_ levels.

Taken together, fertilizer treatments exerted leaf-position-dependent effects on secondary metabolism (Figure 3 and Supplementary Table 4). In B2F leaves, A_27_B_27_ achieved the best combined improvement in phenolic and organic acids; in C3F leaves, A_54_B_54_ displayed the strongest lipophilic and phenolic enhancement, while A_27_B_27_ provided better phenolic balance; and in X2F leaves, A_27_B_54_ realized the highest phenolic acid accumulation with moderate organic acid levels. Overall, mixed fertilization, especially A_27_B_27_ and A_27_B_54_, was more effective in improving phenolic metabolism and chemical coordination, whereas single high-dose treatments (A_0_B_54_ and A_54_B_0_) strongly enhanced certain metabolites but often at the cost of excessive acid load or reduced phenolic accumulation.

### Comprehensive quality evaluation of flue-cured tobacco under different fertilizer regimes

According to the evaluation framework defined by GB/T18771.4-2015, the comprehensive quality of flue-cured tobacco was assessed based on chemical coordination (20%), physical appearance (15%), and sensory quality (60%), with weighted contributions from leaf positions (B2F 0.40, C3F 0.45, X2F 0.15) (Supplementary Table 5). The control treatment (A_0_B_0_) exhibited the lowest overall score (48.39), whereas all fertilizer treatments substantially improved tobacco quality. Among them, the combined application of PGPM at 27 kg⋅ha^–1^ with Xi⋅Weifeng at 54 kg⋅ha^–1^ (A_27_B_54_) achieved the highest overall score (57.70), supported by consistently superior performance across upper (58.08), middle (57.94), and lower leaves (56.57), indicating balanced improvements in chemical composition, appearance, and sensory attributes. Treatments A_27_B_27_ and A_54_B_54_ also performed well, with total scores of 55.38 and 55.93, respectively, highlighting the advantage of co-application strategies. In contrast, sole applications exhibited limited benefits. For instance, A_0_B_54_ improved chemical coordination in middle leaves (13.78 vs. 10.31 in control) but showed lower scores in the lower leaves, leading to only a moderate overall increase (51.32). Similarly, A_54_B_0_ enhanced quality in the middle leaves (53.49) but achieved a relatively low total score of 51.87. Collectively, these findings demonstrate that co-application, particularly A_27_B_54_, provides the most effective improvement in comprehensive tobacco quality across leaf positions, whereas sole fertilizer application tends to produce localized rather than holistic benefits.

### Effects of novel fertilizer concentrations on rhizosphere microbial diversity

**FIGURE 4:**
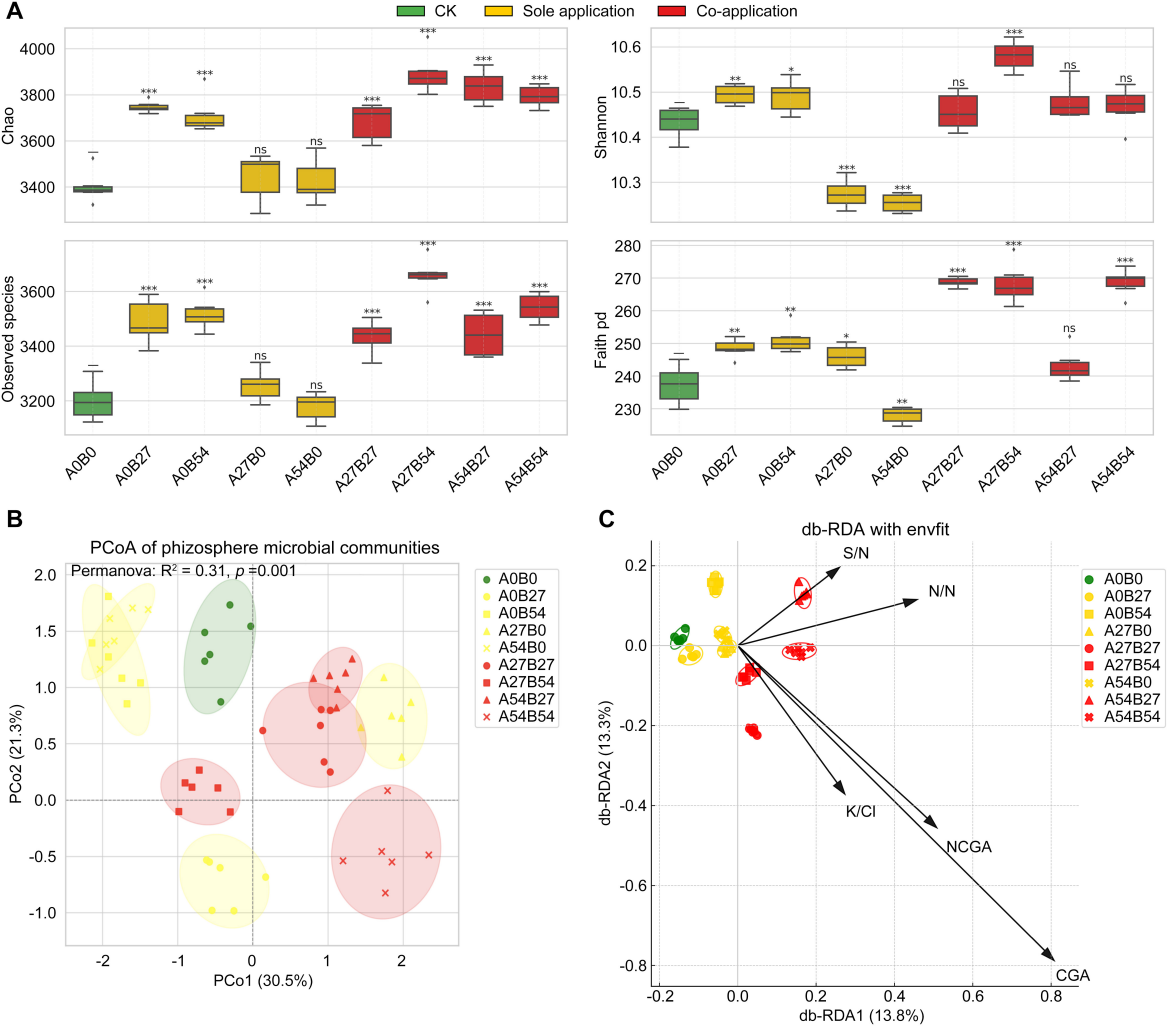
Effects of novel fertilizer application on rhizosphere soil microbial diversity of flue-cured tobacco. **(A)** Boxplots of Chao richness, Shannon diversity, observed species, and Faith’s phylogenetic diversity indices under different treatments. Green indicates the control (A_0_B_0_), yellow represents sole application (A_0_B_27_, A_0_B_54_, A_27_B_0_, A_54_B_0_), and red represents co-application (A_27_B_27_, A_27_B_54_, A_54_B_27_, A_54_B_54_). Significance levels are shown in comparison with the control group (ns, not significant; *p < 0.05; **p < 0.01; ***p < 0.001). **(B)** Principal coordinate analysis (PCoA) based on Bray-Curtis distances, showing differences in community composition across fertilizer treatments. Ellipses represent 95% confidence intervals for each treatment group. PERMANOVA indicated that fertilizer type, dose, and their interaction significantly influenced community composition (overall *R*^2^ = 0.31, *p* = 0.001). **(C)** Distance-based redundancy analysis (db-RDA, capscale) with envfit of rhizosphere bacterial communities constrained by tobacco quality variables. Arrows indicate environmental vectors significantly associated with microbial community variation, with longer arrows representing stronger correlations.

At the flue-curing stage, alpha diversity analysis revealed significant differences in rhizosphere microbial communities among different fertilizer treatments (Figure 4). Compared with the control (A_0_B_0_), most fertilization treatments significantly increased microbial richness and diversity indices. Chao and Observed species were markedly higher under co-application treatments such as A_27_B_54_ and A_54_B_54_, indicating a pronounced enhancement of species richness (Figure 4A). A_27_B_54_ exhibited the highest Chao (3879 ± 95) and observed species (3663 ± 88), significantly surpassing the control (3344 ± 80 and 3182 ± 75, *p* < 0.001). Shannon diversity and Faith’s phylogenetic diversity also showed improvement under co-application (Figure 4A), with A_27_B_54_ again achieving the highest values (10.59 ± 0.05 and 272.5 ± 6.2, *p* < 0.01). In contrast, sole applications (e.g., A_27_B_0_, A_54_B_0_) tended to exert weaker or even suppressive effects on Shannon and Faith’s PD, despite slight increases in richness indices. The results demonstrated that co-application of the two fertilizers was more effective in promoting both taxonomic richness and phylogenetic diversity of rhizosphere microbes than sole application, suggesting a synergistic effect in shaping a more diverse and stable microbial community.

Based on Bray-Curtis distances, a multifactor PERMANOVA (adonis2, 999 permutations) revealed that fertilizer type (control/sole/co-application) was the primary determinant of rhizosphere bacterial community composition, explaining the largest proportion of variance (*R*^2^ = 0.17, *F* = 6.2, *p* = 0.001). Fertilizer dose (0, 27, 54 kg⋅ha^–1^) exerted a secondary effect (*R*^2^ = 0.09, *F* = 3.5, *p* = 0.002), while the interaction between type and dose was also significant (*R*^2^ = 0.05, *F* = 2.1, *p* = 0.010). The overall model explained 30.5% of the variance (*R*^2^ = 0.31), consistent with the separation pattern observed in the PCoA (Figure 4B), indicating that co-application versus sole application generated stable shifts in community structure, with the dosage gradient further modulating these differences. Homogeneity of dispersion tests (betadisper) showed no significant differences in within-group dispersions when samples were grouped by fertilizer type (*F* = 1.21, *p* = 0.22) or by dose (*F* = 1.07, *p* = 0.31). This confirms that the significance of the PERMANOVA results was not driven by heterogeneous dispersions, but by genuine shifts in community centroids. Taken together, the effect hierarchy of fertilizer type >dose >interaction is evident, with co-application emerging as the dominant driver of rhizosphere microbial reassembly.

The db-RDA (capscale) based on Bray-Curtis distances, together with envfit analysis, further elucidated the linkage between microbial community composition and tobacco quality traits (Figure 4C). Fertilizer co-application treatments (e.g., A_27_B_27_, A_27_B_54_, A_54_B_54_) clustered tightly along the positive vectors of CGA, NCGA, and the K/Cl ratio, indicating a strong concordance between microbial shifts and the improvement of key quality indicators. In contrast, sole-application treatments exhibited more dispersed distributions: A_0_B_54_ was strongly aligned with the sugar-to-nicotine ratio (S/N), suggesting an unbalanced increase in sugars relative to alkaloids with insufficient phenolic accumulation, while A_54_B_0_ was more oriented toward the K/Cl vector, reflecting improvements in combustibility but limited effects on phenolic metabolism and compositional coordination. The control (A_0_B_0_) was positioned far from the quality-related vectors, underscoring its overall weaker performance in shaping both community and chemical profiles. Envfit statistics confirmed that CGA (*R*^2^ = 0.29, *p* = 0.002), NCGA (*R*^2^ = 0.24, *p* = 0.004), and K/Cl ratio (*R*^2^ = 0.21, *p* = 0.006) were the strongest predictors of community variation, whereas S/N (*R*^2^ = 0.18, *p* = 0.011) and N/N ratio (*R*^2^ = 0.15, *p* = 0.018) also showed significant but comparatively weaker associations. These results demonstrate that fertilizer co-application not only drives microbial assemblages toward communities associated with enhanced phenolic metabolism and improved combustibility, but also promotes greater chemical coordination. Overall, these results highlighted fertilizer type as the dominant factor mediating both community restructuring and the improvement of tobacco leaf quality traits.

PICRUSt2-based KEGG functional prediction revealed clear differences among fertilization treatments in their ability to restructure the functional potential of root-associated microbial communities. Compared with the control, sole applications of PGPM or Xi⋅Weifeng induced only weak and biologically limited shifts (not significant), indicating that single inoculations exert minimal influence on the core functional architecture of the rhizosphere microbiome. In contrast, all mixed fertilization treatments (A_27_B_27_, A_54_B_27_, A_27_B_54_, A_54_B_54_) produced significant and functionally meaningful alterations across multiple metabolic pathways (Supplementary Figure 3). Specifically, A_54_B_27_ significantly enhanced brassinosteroid biosynthesis and naphthalene degradation. A_27_B_27_ showed marked modulation of the MAPK signaling pathway and proteasome-related functions. A_54_B_54_ exhibited the strongest overall functional shift, with substantial increases in naphthalene degradation, spliceosome-associated functions, and biotin metabolism. The best-performing treatment, A_27_B_54_, displayed the most diverse set of upregulated pathways, including significant enhancement of endocytosis, alongside increased activity in linoleic acid metabolism, flavonoid biosynthesis, and multiple aromatic and xenobiotic degradation pathways (xylene, PAHs, bisphenol), highlighting intensified microbial potential for cross-membrane transport, lipid and phenolic metabolism, and the transformation of complex organic substrates. Collectively, these results demonstrate that mixed fertilization induces a multi-dimensional reprogramming of microbial functional capacity, particularly strengthening hormone-related metabolism, membrane transport, aromatic substrate degradation, and pathways associated with phenolic and lipid metabolism, thereby providing mechanistic support for the observed improvements in tobacco growth, chemical coordination, and aroma-related quality traits.

### Differential responses of rhizosphere microbial communities to fertilizer treatments and their associations with tobacco traits

At the maturation stage, the rhizosphere microbial community of tobacco exhibited pronounced shifts under different fertilizer treatments (Supplementary Figure 4). In the control treatment (A_0_B_0_), the community was dominated by Acidobacteriota, *Gemmatimonas*, and *Sphingomonas*, reflecting the prevalence of oligotrophic taxa. With the application of PGPM and Xi⋅Weifeng, either singly or in combination, the relative abundances of several key genera changed markedly. In general, the restructuring effect on dominant taxa was more evident under co-application, where *Bacillus*, *Burkholderia-Caballeronia-Paraburkholderia* (BCP), and *Sphingomonas* showed consistently higher abundances compared with the control and sole application. Specifically, BCP reached the highest level in treatment A_27_B_54_, *Bacillus* was most enriched in A_54_B_54_ and A_27_B_27_, while *Sphingomonas* increased most prominently in A_54_B_27_.

**FIGURE 5:**
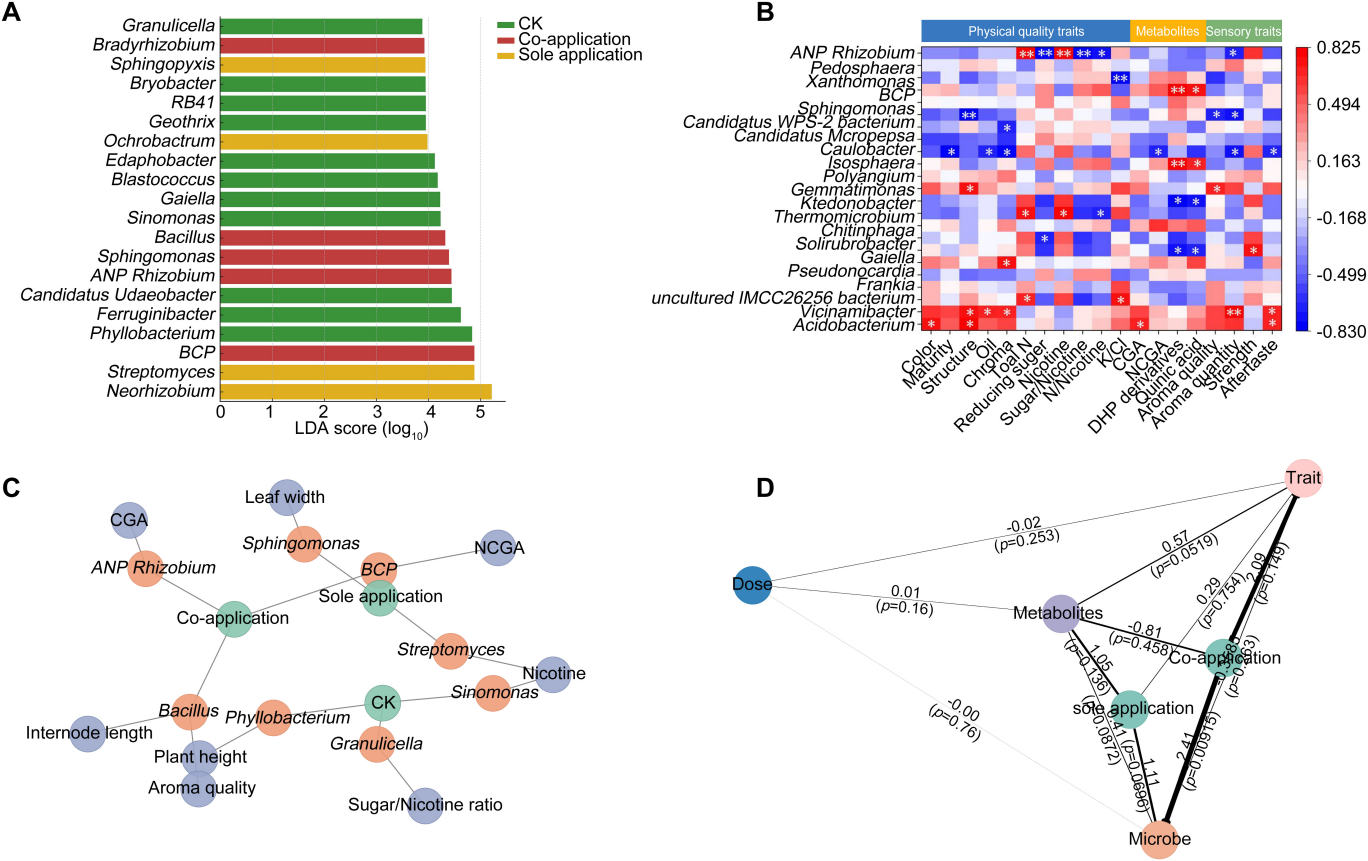
Integrative analysis of fertilization effects on rhizosphere microbiota, metabolite coordination, and tobacco quality traits. **(A)** LEfSe analysis showing discriminant microbial biomarkers (LDA score > 4.0, *p* < 0.05). **(B)** Heatmap of Spearman correlations between representative genera and agronomic/chemical traits (red = positive, blue = negative; *p < 0.05; **p < 0.01). **(C)** Network integrating fertilizer treatments, microbial biomarkers, and traits, with nodes representing treatments (green), genera (orange), and traits (blue). **(D)** Structural equation model (SEM) linking fertilizer inputs (dose and application type), rhizosphere microbial community, metabolites, and agronomic/quality traits in flue-cured tobacco. Path coefficients are shown along the arrows, with *p*-values in parentheses. Arrow thickness reflects effect size, and significant pathways (*p* < 0.05) are highlighted in bold.

Linear discriminant analysis effect size analysis further identified distinct microbial biomarkers across treatments (Figure 5A). In the control group, oligotrophic Acidobacteria genera such as *Granulicella*, *RB41*, and *Edaphobacter* were significantly enriched, indicating that the community structure was largely shaped by indigenous taxa adapted to nutrient-poor environments in the absence of exogenous microbial inputs. Sole application was characterized by the enrichment of genera such as *Sphingopyxis*, *Geothrix*, and *Streptomyces* (LDA > 4.2, *p* < 0.05). By contrast, co-application significantly enriched a suite of taxa with known plant growth-promoting and nutrient-cycling capacities. In particular, *Allorhizobium*-*Neorhizobium*-*Pararhizobium*-*Rhizobium* (LDA = 4.8), *Burkholderia-Caballeronia-Paraburkholderia* (LDA = 4.7), *Bacillus* (LDA = 4.5), and *Sphingomonas* (LDA = 4.3) emerged as robust biomarkers under co-application.

Correlation analysis revealed significant associations between rhizosphere microbial taxa and multiple agronomic and chemical traits of tobacco (Figure 5B). For example, *Allorhizobium*-*Neorhizobium*-*Pararhizobium*-*Rhizobium* and *Sphingomonas* were positively correlated with nicotine content, while *Burkholderia-Caballeronia-Paraburkholderia* showed significant associations with nitrogen-related metabolites and leaf width. Conversely, *Acidobacterium* and *Vicinamibacter* were negatively correlated with aroma quality and sugar-related traits. Importantly, co-application-enriched genera such as *Bacillus* and *Phyllobacterium* exhibited positive correlations with plant height and aroma quality, suggesting that these microbes may contribute to both vegetative growth and improvements in leaf quality.

The integration of fertilizer treatments, microbial biomarkers, and agronomic traits through network analysis provided further insights into these associations (Figure 5C). The network topology revealed three distinct clustering patterns: (i) co-application was linked to Bacillus and *Allorhizobium*-*Neorhizobium*-*Pararhizobium*-*Rhizobium*, which were further connected with plant height, aroma quality, and CGA content; (ii) sole application was mainly connected to *Burkholderia-Caballeronia-Paraburkholderia* and *Streptomyces*, which showed strong associations with nicotine and nitrogenous compounds; and (iii) the control treatment was associated with *Granulicella* and *Sinomonas*, correlating with the sugar/nicotine ratio and nicotine content, respectively. Overall, the microbial–trait network under co-application was more conducive to plant growth and leaf quality enhancement, whereas sole application and the control were more closely related to traits indicative of unfavorable or neutral quality outcomes. These findings demonstrate that fertilization regimes shape rhizosphere microbial communities, which in turn regulate tobacco quality traits through specific microbial-plant associations.

The structural equation modeling (SEM) analysis revealed that fertilization exerted the strongest effect on rhizosphere microbial communities (Figure 5D). In particular, co-fertilization significantly increased microbial PC1 scores (coef = 2.41, *p* = 0.009), whereas sole fertilization showed a marginally positive effect (coef = 1.11, *p* = 0.070). By contrast, fertilizer dose did not significantly influence microbial composition (*p* = 0.76). Changes in microbial communities further exhibited a marginally significant positive effect on metabolite profiles (coef = 0.41, *p* = 0.087), suggesting that microbiome restructuring may be a key driver of metabolic reprogramming. At the trait level, metabolite PC1 was positively associated with agronomic and quality traits (coef = 0.57, *p* = 0.052), whereas direct effects of microbes or dose on traits were not significant. Collectively, these results support an indirect pathway in which fertilization type first alters the microbial community, which in turn modulates metabolite profiles, ultimately influencing plant traits. This indicates that mixed fertilization improves plant performance by reshaping the rhizosphere microbiota and subsequently regulating secondary metabolism.

## Discussion

Compared with recent studies that have primarily focused on single PGPR/PGPF strains or single commercial microbial fertilizers, the present study is distinguished by its dual-inoculant design integrating a self-formulated PGPM consortium based on *B. subtilis* and *B. licheniformis* together with a commercial microbial fertilizer (Xi⋅Weifeng), implemented across a two-factor “type × dose” gradient that enabled systematic comparison of sole versus combined inputs. By coupling this factorial design with agronomic traits, chemical quality indicators, key aroma- and combustibility-related metabolites, 16S rRNA sequencing, multivariate analyses, and structural equation modeling, we aimed to address a deeper mechanistic question rarely examined in tobacco systems, which was how microbial fertilization reshapes rhizosphere community structure and downstream metabolic pathways to modulate flue-cured tobacco quality. Our preliminary pot experiment provided important mechanistic clues. Under sterile conditions, typical PGPM metabolites such as IAA, ACC deaminase, and lipopeptides failed to promote shoot growth. However, both *Bacillus* strains markedly enhanced plant stature and root development when plants were grown in soil. This contrast supports the emerging view that growth promotion does not arise from direct metabolite activity but instead depends on successful microbial colonization and interactions within the native rhizosphere microbiome. Similar conclusions were reported in tobacco by Shi et al. (2024) and in other crops by Chai et al. (2024), who demonstrated that PGPR and PGPF efficacy relies on host-microbe-soil tripartite interactions rather than isolated bioactive compounds. Mechanistically, our approach aligns with the rapidly expanding papers integrating microbial inoculants with metabolomics (e.g., Das and Maity, 2025; Yuan et al., 2023), but advances the field by linking microbiome restructuring, metabolic pathway shifts (CGA/NCGA, organic acids, lipophilic fractions), chemical coordination indices, and agronomic/quality outcomes within a unified causal framework.

Building on this mechanistic foundation, our field results further demonstrate that the dual-microbial co-application strategy proposed here confers distinct agronomic and quality advantages. Mixed treatments promoted plant height and leaf expansion at developmentally responsive stages while avoiding the inhibitory effects of high-dose single inputs; shifted sugar/nicotine and nitrogen/nicotine ratios toward the ideal coordination zone across all leaf positions; and selectively enriched functionally important genera associated with nutrient turnover, phenolic biosynthesis, and aroma formation, which were positively correlated with CGA accumulation, aroma attributes, and plant stature. Ultimately, SEM revealed that fertilization “type,” rather than “dose,” was the principal driver of phenotypic improvement, acting through a sequential mechanism in which the fertilization regime first restructured the rhizosphere microbiome, subsequently altered the metabolic profile of the plant, and finally led to measurable enhancements in agronomic performance and leaf quality. This integrative mechanism has been proposed conceptually in multi-omics studies of other crops but, to our knowledge, is reported here for the first time in a tobacco quality context with comprehensive evidence spanning microbial, metabolic, and phenotypic layers.

Despite these strengths, several limitations should be acknowledged, particularly in light of the mechanistic themes explored in the following sections. First, the field experiment was conducted at a single site within one growing season, and the performance of microbial inoculants is known to be highly context-dependent across soil conditions, climates, and native microbiome backgrounds. This constrains the generalizability of our conclusions and highlights the need for future multi-location, multi-year validation to assess whether the complementary functional niches, metabolite coordination patterns, and microbiome-trait linkages identified here are robust across agroecosystems. Second, although the integration of 16S profiling, metabolic assays, and structural equation modeling enabled us to propose mechanistic pathways linking fertilization regimes to quality formation, the regulatory basis at the transcriptional level remains unresolved. Incorporating root and leaf transcriptomics along with metatranscriptomic profiling of the rhizosphere microbiome will be essential for revealing how microbial inputs reprogram plant and microbial pathways in a coordinated manner.

### Mixed fertilization confers complementary functional niches that stabilize plant performance

Why does co-application outperform single inputs? A common explanation is functional complementarity among microbial consortia that broadens niche coverage in the rhizosphere and buffers plants against resource or environmental variability. Recent studies have demonstrated that microbial consortia generally exhibit higher ecological adaptability and functional redundancy than single strains in complex soil environments, enabling them to maintain stable growth-promoting effects under resource fluctuations or environmental stress (Hossain et al., 2025; Mashabela et al., 2022). Consistent with this evidence, the advantage of mixed fertilization observed in our study does not arise from the simple additive effects of individual microbes, but from a multi-pathway functional complementarity operating in parallel. Typical PGPM consortia frequently combine phytohormone modulation (IAA, cytokinin), ACC-deaminase-mediated ethylene dampening, and siderophore/phosphate-solubilization functions (Jose et al., 2025). Within these consortia, *Bacillus* contribute robust spore-based persistence, rhizosphere competence, and lipopeptide-mediated ISR (induced systemic resistance) (Choudhary and Johri, 2009); BCP (*Burkholderia-Caballeronia-Paraburkholderia*) includes diazotrophs and aromatic-compound degraders that can accelerate N cycling and carbon flow (Caballero-Mellado et al., 2007; Vanwijnsberghe et al., 2021); *Rhizobiales* taxa engage in N fixation and signaling cross-talk with roots (Lindström and Mousavi, 2020; Masson-Boivin and Sachs, 2018), and *Sphingomonas* contributes stress tolerance and xenobiotic turnover (Innerebner et al., 2011). The LEfSe biomarkers identified in this study align closely with these functional attributes, and network analyses further reveal that, under mixed fertilization, the co-enrichment of these functional groups is strongly associated with plant height, CGA levels, and aroma-related traits. These patterns indicate that “functional complementarity” within the microbiome ultimately manifests as physiological complementarity at the plant level.

Notably, recent multi-omics studies have demonstrated that the mode of microbial input, such as fertilization type or whether products are co-applied exerts a far stronger influence on microbial network restructuring than the intensity of input, particularly in systems where microbial amendments can trigger rhizosphere self-modulation (Nie et al., 2025; Yang H. et al., 2025). Consistent with these findings, the effect hierarchy observed in our study, where PERMANOVA hierarchy (type > dose) and PCoA separations jointly indicate that the primary determinant of rhizosphere community organization is not the absolute amount of microbial or nutrient input, but rather the interaction, competition, and coexistence dynamics established between the introduced microbes and the native rhizosphere consortium. This mode-driven assembly process enhances community stability, increases functional redundancy, and amplifies downstream metabolic consequences, ultimately strengthening chemical coordination, activating CGA/NCGA-related pathways, and improving overall quality traits.

Taken together, mixed fertilization does not act by simply increasing microbial abundance; instead, it promotes systemic improvement in tobacco quality through a multilayered mechanism involving rhizosphere microenvironment modulation, selective microbial recruitment, functional complementarity, and metabolic pathway reprogramming (Li et al., 2017; Yan et al., 2024). To our knowledge, this work is the first to systematically reveal how dual-microbial-fertilizer co-application generates cascading reinforcement across ecological niches, microbiome structure, and metabolic networks in the tobacco system, thereby providing a mechanistic framework for precision formulation of future microbial fertilizers.

### Chemical coordination as the proximate phenotype linking microbes to quality

The improved chemical coordination under co-fertilization provides a proximate explanation for the enhanced quality scores (convergence toward the accepted sugar/nicotine (8–12) and N/nicotine (0.7–1.0) ranges), and mechanistically this outcome may be attributed to three interrelated routes by which microbiome shifts alter leaf chemistry. First, nutrient partitioning, where diazotrophic and P-mobilizing taxa elevate root uptake efficiency and adjust source–sink dynamics, thereby moderating nicotine synthesis (a nitrogen-rich alkaloid) while supporting soluble sugars and organic acids at balanced levels (Ipsilantis et al., 2018; Pang et al., 2024); second, hormonal signaling, as PGPR-derived IAA and ACC-deaminase reduce stress ethylene, promoting leaf expansion (such as wider leaves at maturity) and potentially reallocating carbon toward phenylpropanoids (e.g., CGAs and NCGAs) that are crucial for aroma and combustibility (Glick, 2014; Pieterse et al., 2014); and third, phenylpropanoid pathway priming, in which root-microbe interactions activate PAL/4CL/CHS nodes to increase phenolic pools that underpin aroma complexity and oxidative stability during curing (Van Wees et al., 2008; Zamioudis and Pieterse, 2012). Notably, our db-RDA revealed that community axes aligned with CGA/NCGA and K/Cl vectors under co-application, a pattern consistent with coordinated improvements in combustibility and flavor.

Beyond these three routes, recent multi-omics studies further support that rhizosphere microbes can reconfigure central carbon-nitrogen metabolism and channel metabolic flux into specific secondary pathways that determine leaf quality (Liu et al., 2025; Yang T. et al., 2025). For example, integrated microbiome-metabolome analyses in tobacco have shown that shifts in bacterial consortia are tightly coupled to changes in organic acids, phenolic acids and nitrogenous metabolites that define aroma style and burning characteristics, highlighting a direct linkage between microbiome composition and leaf metabolic states (Yu et al., 2023). Likewise, plant growth-promoting rhizobacteria in other crops have been shown to remodel primary nitrogen assimilation and associated secondary metabolism, increasing amino acid pools while enhancing phenylpropanoid and flavonoid biosynthesis, which in turn strengthens both defense and quality-related traits (Chi et al., 2025; Das and Maity, 2025). These findings are in line with broader evidence that microbial inoculants systematically elevate the accumulation of bioactive phenolics, chlorogenic acids and antioxidant compounds through coordinated modulation of hormone signaling, redox status and carbon skeleton supply. Taken together, our results fit into this emerging framework by showing that co-fertilization not only improves classical coordination indices (sugar/nicotine and N/nicotine), but also steers phenylpropanoid and organic-acid centered pathways toward a metabolic configuration that favors balanced combustibility and richer aroma, thereby providing a mechanistic bridge from rhizosphere microbial restructuring to integrative quality formation in flue-cured tobacco.

### Network and pathway evidence linking microbial fertilization to tobacco quality

The network analysis integrated fertilizer regimes, microbial biomarkers, and phenotypic traits, revealing three distinct modules: the co-application module (*Bacillus* and *Rhizobiales* linked to plant height, CGAs, and aroma quality), the sole-application module (*BCP* and *Streptomyces* associated with nicotine and nitrogenous compounds), and the control module (*Granulicella* and *Sinomonas* correlated with the sugar/nicotine ratio). This modular structure is consistent with previous reports on disease-suppressive soils and plant growth-promoting microbial assemblages, where keystone taxa sustain host traits through cross-feeding and niche partitioning (Mendes et al., 2013; Toju et al., 2018). Notably, traits aggregated in the co-application module were more integrative (aroma, CGAs, and plant stature) rather than single indicators, implying those dominant taxa function as system-level integrators in rhizosphere networks.

The structural equation model further quantified causal hierarchies, showing that fertilization type exerted strong effects on rhizosphere communities, which in turn shaped metabolite profiles, ultimately explaining significant variation in agronomic and quality traits. In contrast, fertilizer dose and direct microbial effects on traits were weak or non-significant. This aligns with the broader conceptual framework of “from management practices to rhizosphere communities to plant metabolism to crop phenotypes” (de Vries et al., 2018; Trivedi et al., 2020). Importantly, our results highlight the role of indirect effects, suggesting that designing microbial consortia with complementary functions and stable colonization capacities is more effective than increasing inputs alone. From an agronomic perspective, this finding emphasizes the potential of functional consortia and staged application strategies to guide plant metabolism toward coordinated quality outcomes. Overall, our findings provided two applied implications. First, moderate co-application of microbial fertilizers is the most effective strategy to enhance flue-cured tobacco quality, as it reshapes beneficial communities while avoiding the inhibitory effects observed at higher doses. Second, quality diagnostics should integrate microbial biomarkers (e.g., *Bacillus*, *BCP*, *Rhizobiales*, *Sphingomonas*) with metabolic indicators (CGA/NCGAs, K/Cl ratio) rather than relying on single chemical traits.

## Conclusion

This study demonstrates that co-application of a *Bacillus*-based PGPM and a commercial microbial fertilizer provides synergistic benefits for flue-cured tobacco by integrating plant growth responses, leaf chemical coordination, and rhizosphere microbiome restructuring. Preliminary pot experiments showed that *B. subtilis* and *B. licheniformis* enhanced plant height, stem robustness, and root development only in soil-grown plants, indicating that growth promotion depends on colonization and interactions with the native microbiome rather than direct metabolite effects. Field results further revealed that moderate PGPM application consistently improved plant growth performance, while mixed fertilization shifted sugar/nicotine and nitrogen/nicotine ratios toward ideal coordination ranges and increased phenolic acids, organic acids, and lipophilic components across leaf positions, resulting in the highest comprehensive quality scores. Microbiome analyses showed that fertilization “type,” rather than “dose,” was the primary driver of community restructuring, with co-application enriching key functional genera linked to nutrient turnover, phenylpropanoid metabolism, combustibility traits, and aroma formation. Network analysis and SEM confirmed a sequential mechanism in which the fertilization regime reshapes the rhizosphere microbiome, reprograms leaf metabolic profiles, and ultimately improves agronomic and quality traits. Overall, this work provides integrated microbial, metabolic, and phenotypic evidence that mixed microbial fertilization represents a robust, mechanism-based strategy for enhancing tobacco quality and offers a framework for the precision design of future microbial fertilizer formulations.

## Data Availability

The datasets presented in this study can be found in online repositories. The names of the repository/repositories and accession number(s) can be found below: https://www.ncbi.nlm.nih.gov/, PRJNA1333626.

## Footnotes

1
http://huttenhower.sph.harvard.edu/galaxy/

## Supplementary material

Supplementary Figure 1 Root morphological responses of tobacco to single inoculation with *Bacillus licheniformis* and *B. subtilis* in the preliminary pot experiment.

Supplementary Figure 2 Effects of different fertilization treatments on leaf number, stem girth, and leaf length of flue-cured tobacco.

Supplementary Figure 3 Relative abundances of the top 10 genera across treatments.

Supplementary Figure 4 Differential volcano plots of predicted microbial functional pathways (PICRUSt2) under mixed fertilization treatments compared with the control (A_0_B_0_).

Supplementary Table 1 Concentrations of representative PGPM-related metabolites in the self-prepared PGPM fertilizer and the commercial product Xi⋅Weifeng.

Supplementary Table 2 Differences in agronomic traits of tobacco plants among different treatments.

Supplementary Table 3 Effect of different treatments on the chemical composition of various parts of tobacco leaves.

Supplementary Table 4 Effect of different treatments on secondary metabolites in various parts of tobacco leaves.

Supplementary Table 5 Comprehensive quality analysis of flue-cured tobacco among different treatments.
