# Supplementary figures and images for: Mixed application of microbial fertilizers reshapes the tobacco rhizosphere microbiome and enhances metabolic coordination to improve crop quality
Source: Front Microbiol. 2026 Jan 15;16:1726681. doi: 10.3389/fmicb.2025.1726681 (PMC12852418; doi:10.3389/fmicb.2025.1726681)

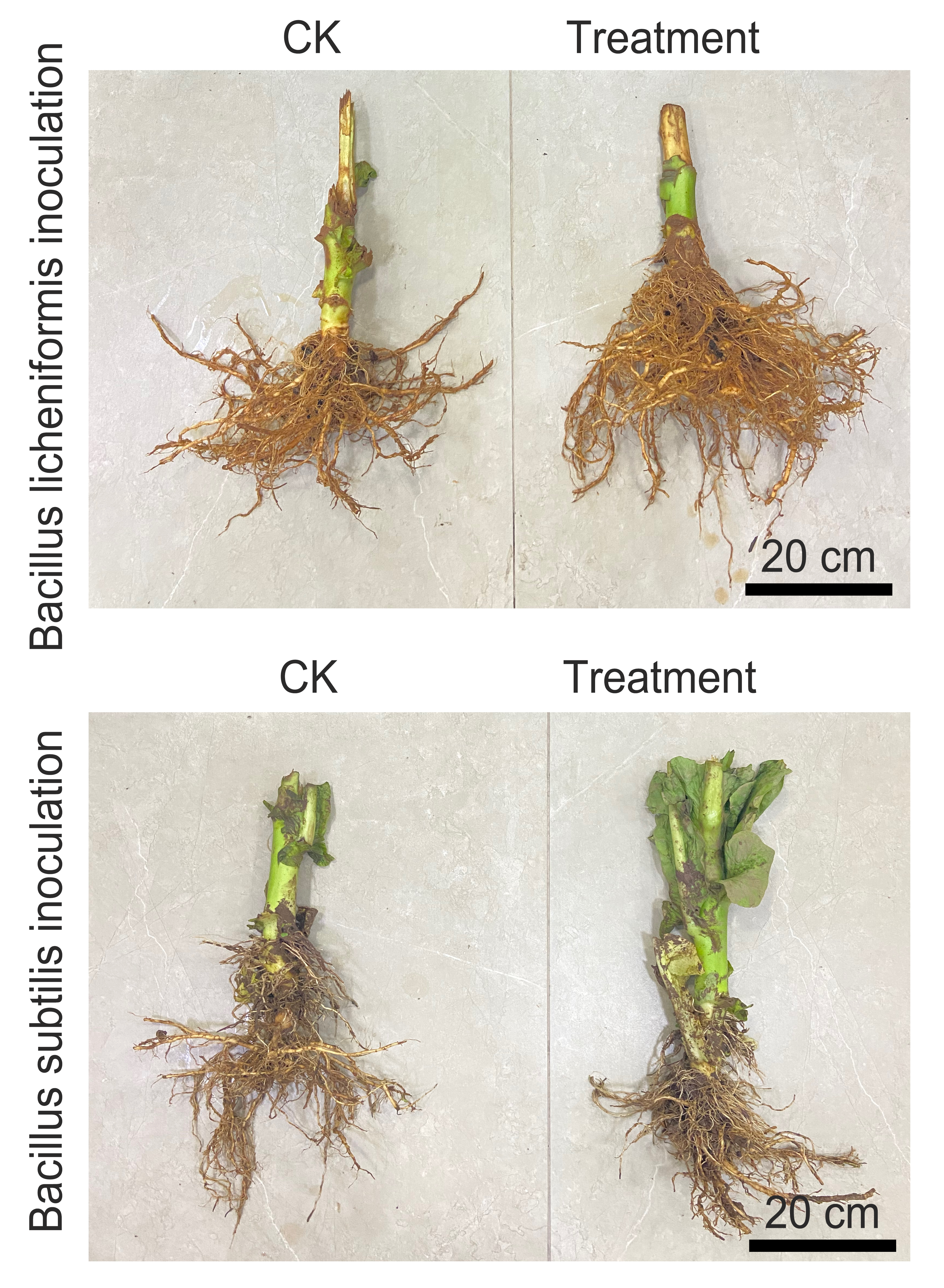

Supplement: Supplementary Figure 1 — Root morphological responses of tobacco to single inoculation with Bacillus licheniformis and B. subtilis in the preliminary pot experiment. [file Image_1.tif]

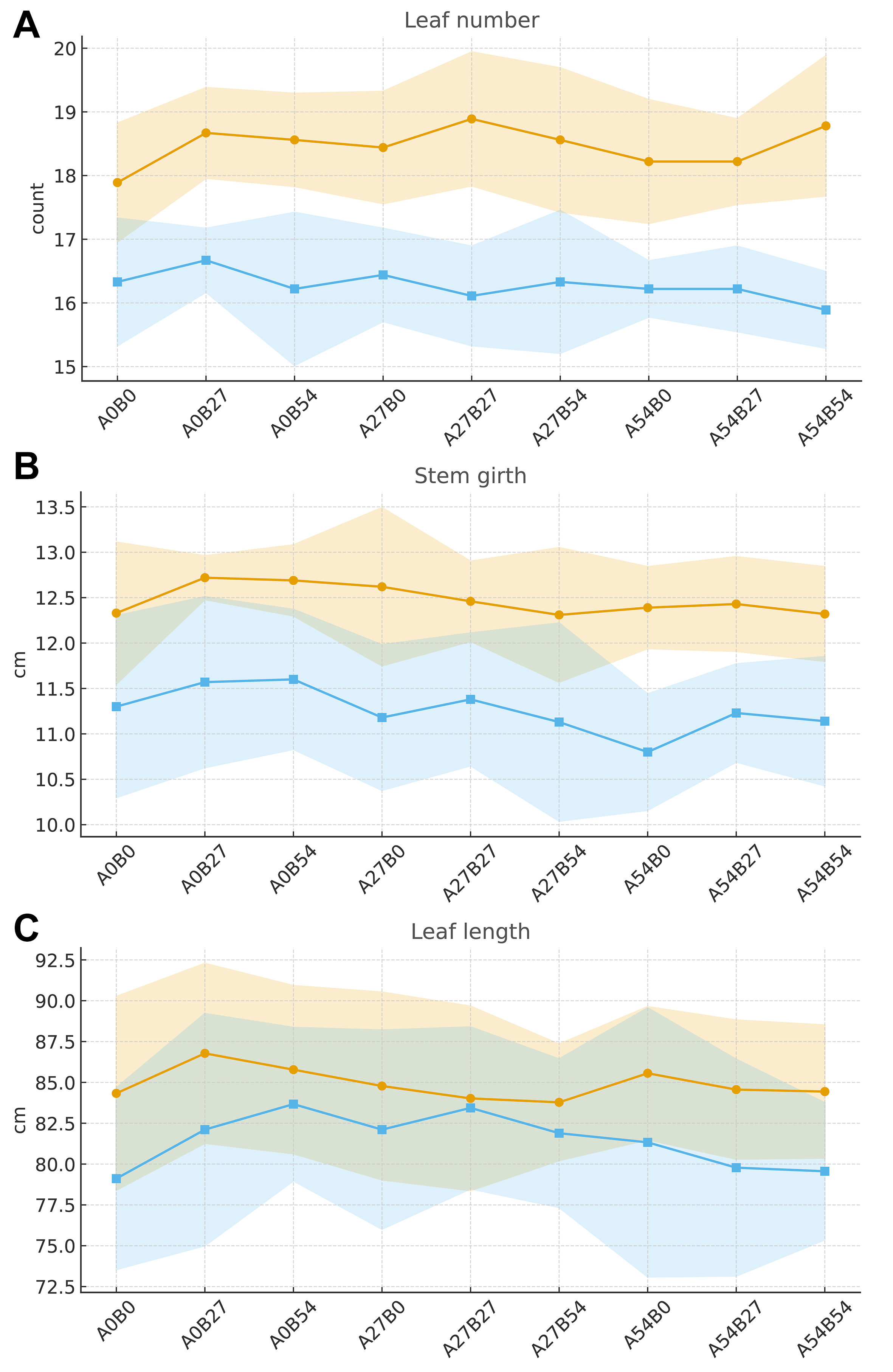

Supplement: Supplementary Figure 2 — Effects of different fertilization treatments on leaf number, stem girth, and leaf length of flue-cured tobacco. [file Image_2.tif]

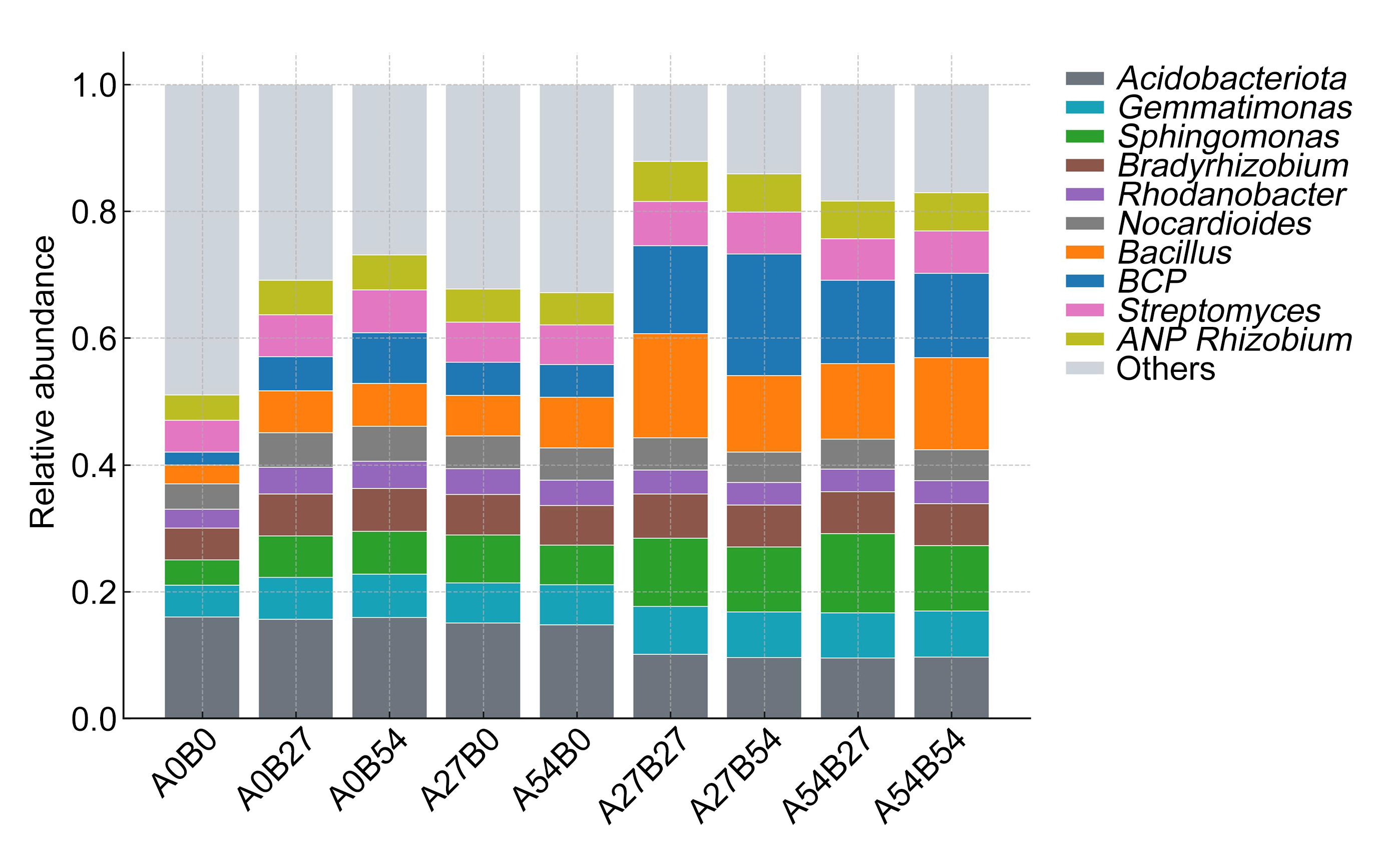

Supplement: Supplementary Figure 3 — Relative abundances of the top 10 genera across treatments. [file Image_3.tif]

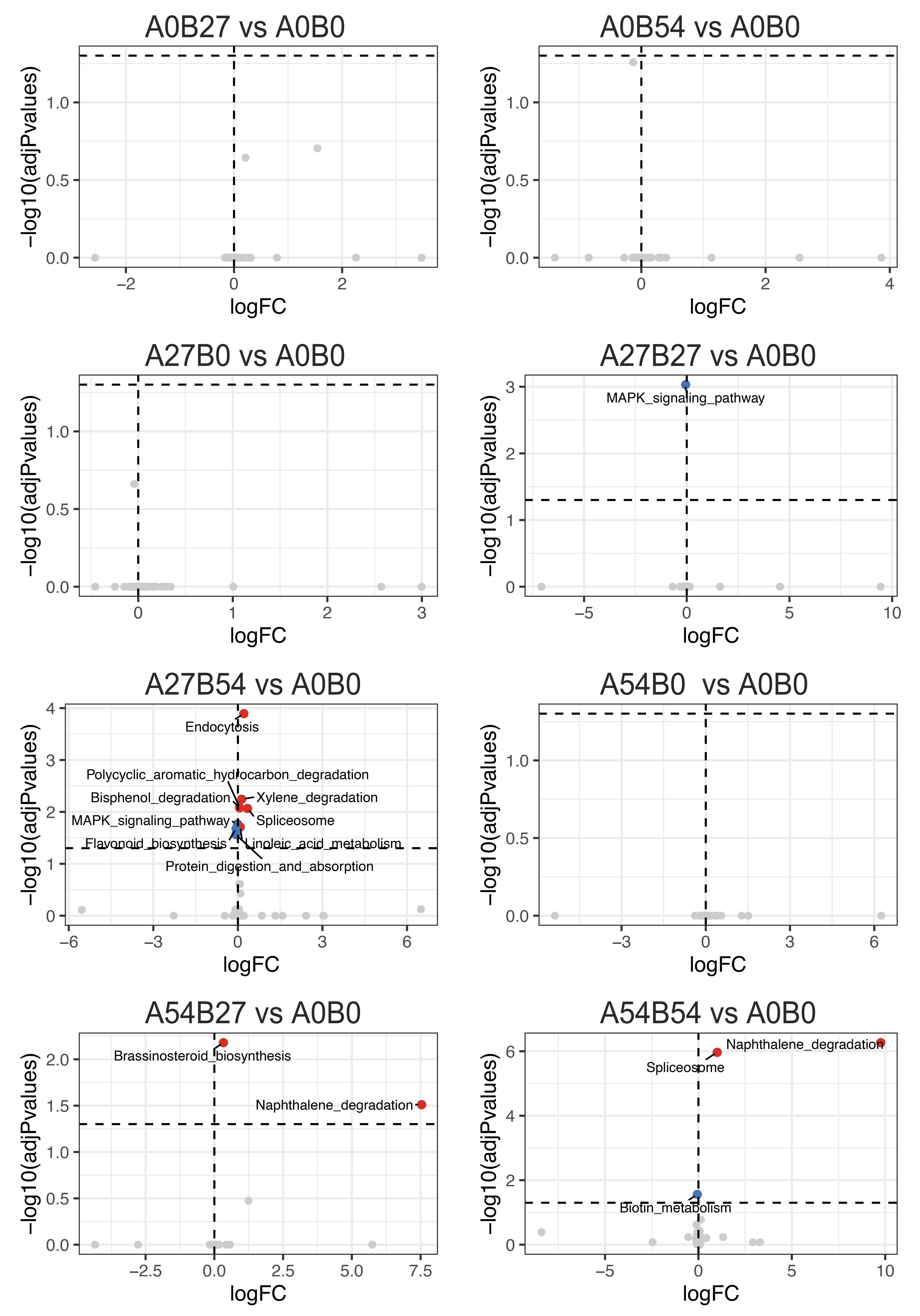

Supplement: Supplementary Figure 4 — Differential volcano plots of predicted microbial functional pathways (PICRUSt2) under mixed fertilization treatments compared with the control (A0B0). [file Image_4.tif]
